# Structural differences of amyloid-β fibrils revealed by antibodies from phage display

**DOI:** 10.1186/s12896-015-0146-8

**Published:** 2015-06-18

**Authors:** Patrick Droste, André Frenzel, Miriam Steinwand, Thibaut Pelat, Philippe Thullier, Michael Hust, Hilal Lashuel, Stefan Dübel

**Affiliations:** Technische Universität Braunschweig, Institute of Biochemistry, Biotechnology and Bioinformatics, Spielmannstr.7, 38106 Braunschweig, Germany; Current address: Celerion Switzerland AG, Allmendstrasse 32, 8320 Fehraltorf, Switzerland; YUMAB GmbH, Rebenring 33, 38106 Braunschweig, Germany; Current address: Delenex Therapeutics AG, Wagistrasse 27, 8952 Schlieren, Switzerland; Institut de recherche Biomédicale des Armées (IRBA-CRSSA); Département de Microbiologie; Unité de biotechnologie des anticorps et des toxines, La Tronche Cedex, France; Current address: BIOTEM Parc d’Activités Bièvre Dauphine, 885, rue Alphonse Gourju, 38140 Apprieu, France; SV-BMI, Laboratory of Molecular and Chemical Biology of Neurodegeneration, Brain Mind Institute, École Polytechnique Fédérale de Lausanne, Station 19, 1015 Lausanne, Switzerland

**Keywords:** Alzheimer’s disease, Aβ, Abeta, Beta-amyloid, Phage display, Immune library, scFv

## Abstract

**Background:**

Beside neurofibrillary tangles, amyloid plaques are the major histological hallmarks of Alzheimer’s disease (AD) being composed of aggregated fibrils of β-amyloid (Aβ). During the underlying fibrillogenic pathway, starting from a surplus of soluble Aβ and leading to mature fibrils, multiple conformations of this peptide appear, including oligomers of various shapes and sizes. To further investigate the fibrillization of β-amyloid and to have tools at hand to monitor the distribution of aggregates in the brain or even act as disease modulators, it is essential to develop highly sensitive antibodies that can discriminate between diverse aggregates of Aβ.

**Results:**

Here we report the generation and characterization of a variety of amyloid-β specific human and human-like antibodies. Distinct fractions of monomers and oligomers of various sizes were separated by size exclusion chromatography (SEC) from Aβ42 peptides. These antigens were used for the generation of two Aβ42 specific immune scFv phage display libraries from macaque (*Macaca fascicularis*). Screening of these libraries as well as two naïve human phage display libraries resulted in multiple unique binders specific for amyloid-β. Three of the obtained antibodies target the N-terminal part of Aβ42 although with varying epitopes, while another scFv binds to the α-helical central region of the peptide. The affinities of the antibodies to various Aβ42 aggregates as well as their ability to interfere with fibril formation and disaggregation of preformed fibrils were determined. Most significantly, one of the scFv is fibril-specific and can discriminate between two different fibril forms resulting from variations in the acidity of the milieu during fibrillogenesis.

**Conclusion:**

We demonstrated that the approach of animal immunization and subsequent phage display based antibody selection is applicable to generate highly specific anti β-amyloid scFvs that are capable of accurately discriminating between minute conformational differences.

## Background

Alzheimer’s Disease is a slowly progressing, irreversible neurodegenerative disorder and the most prevalent cause of dementia in the elderly. With 7.7 million new cases every year and a survival time after diagnose of 7.1 years [1] the number of over 35 million people suffering as of 2012 is thought to be tripled by the year 2050 according to the world health organization (WHO). Accompanied by this, the annual cost generated by dementia, currently exceeding 600 billion $, will most likely rise to more than 1,100 billion $ within the next 15 years. It is the socioeconomic impact which lays the foundation for the urgent need of diagnostic and therapeutic tools in AD that target the disease and its progression at an early stage.

Histological hallmarks of AD are neurofibrillary tangles, comprised of hyperphosphorylated tau protein [2,3], and amyloid plaques that are composed of aggregated amyloid-β peptides [4-6]. Amyloid-β is regarded as the main culprit causing the neuropathology in AD and is released from the amyloid precursor protein by sequential cleavage with β- and γ-secretases. This processing results in peptides of various amino acids (aa) in length with the majority being 40 aa (90%) and 42 aa (10%) long [7], hence the terminology Aβ40/42. Changes in the metabolism of Aβ lead to an imbalance between elevated peptide production and decreased clearance from the brain, shifting the concentration and facilitating self aggregation of β-amyloid. Once a critical concentration is surpassed, the aggregation follows a nucleation-dependent polymerization process to form mature fibrils with various oligomeric intermediates along the way [8,9]. A multitude of diverse Aβ aggregates has been identified, such as dimers [10,11], heteromorphous oligomers [12-16], or protofibrils [17], that represent the last stage before the final transition into the fibril forms. Oligomers and protofibrils are widely regarded as the main toxic species in AD although the exact nature of the toxic entity - if such a form even exists [18] - has yet to be elucidated [19-25].

While on the one side researchers investigating how Aβ contributes its toxicity to AD there are still other problems close at hand: up until today it is neither possible to diagnose the disease at an early, presymptomatic stage nor to treat patients beyond symptomatic relief, e.g. alleviating behavioral problems. The first symptoms emerge decades after neuronal changes occur [26]. Therefore the current diagnoses target progressed characteristics of the disease and are composed of various imaging methods such as x-ray computed tomography (CT) succeeded by magnetic resonance imaging (MRI) [27,28] or positron emission tomography (PET) [29], additional to cognitive tests and the assessment of the patient’s history regarding the worsening of cognition. Still, the combination of these tools does not result in an absolute accuracy of the diagnosis [30]. Additionally, to modify the progression of Alzheimer’s disease it is essential to apply potential therapies at an early stage, long before amyloid plaques are formed [31]. Current treatment of AD involves acetylcholinesterase inhibitors (e.g. Donepezil) [32,33] and N-methyl-D-aspartate (NMDA) antagonists [34] to improve cognitive functionality, up until now only with remote success. For an early and accurate diagnosis of the disease as well as for a better treatment hypothesis, it is essential to get a deeper insight on the aggregation of amyloid-β.

During the transition from Aβ monomer to fibrils, different conformational epitopes are expected to form, which may be used to differentiate between diverse aggregation forms of Aβ using antibodies specifically recognizing these conformational epitopes. Phage display and immune libraries from macaque have been proven in the past to be an effective instrument for the generation of conformation specific antibodies, already providing a source of binders against targets like ricin [35], anthrax [36,37], bacterial surface proteins such as Crf2 from *Aspergillus fumigatus* [38], the Venezuelan equine encephalitis virus (VEEV) [39] and the western equine encephalitis virus (WEEV) [40] or botulinum neurotoxin A [41]. A further distinct advantage of NHP (non human primate) derived immune libraries is the very high degree of identity of the antibodies to human antibodies [42] allowing for very easy transition of the scFvs from diagnostic to therapeutic tools. Phage display antibody generation further allows to control the conditions and conformations during the very moment of binder selection, offering additional chances to steer antibody specificity towards conformational epitopes [43].

## Results

### Antigen preparation (Aβ42)

**Figure 1:**
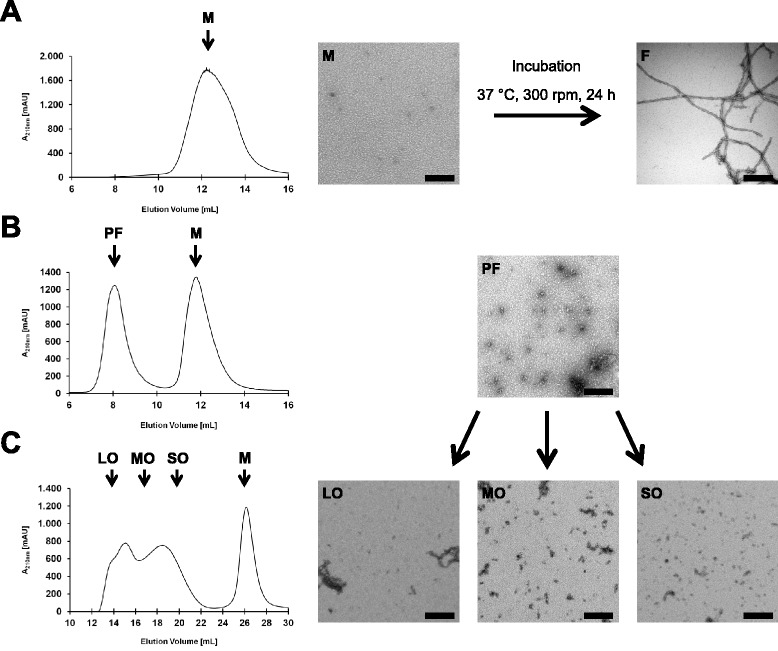
Generation of Aβ42 fractions. Aβ42 peptide was solubilized in 6 M Guanidin-HCl (for sole monomer preparation) or by DMSO, dH_2_O and Tris (for protofibril preparation) and purified by SEC. **(A)** single column purification of a sole monomer (M) fraction. Fibrils (F) were derived from monomers by incubation at 37°C, **(B)** single column purification of a monomer (M) and protofibrils (PF) fraction, **(C)** purification via two columns connected in series to further separate the protofibrils fraction and obtain large oligomers (LO), medium oligomers (MO) or small oligomers (SO). Left: SEC chromatograms, right: representative TEM images, scale bar corresponds to 200 nm.

Fractions of Aβ42 monomers, protofibrils and mature fibrils were prepared from synthetic Aβ42 peptide to serve as antigens. Depending on the purification method, the separation via SEC with one column resulted either solely in pure monomers (Figure 1A) or a monomer fraction and a second peak representing a heterogeneous mixture of different sized oligomers, namely protofibrils (Figure 1B). These protofibrils range between 15 kDa and 500 kDa and display various forms and morphologies, with diameters of 8–10 nm and a length of up to 200 nm. Protofibrils were further separated by two SEC columns connected in series to obtain smaller or larger oligomers (Figure 1C). Earlier eluting fractions include filaments significantly larger than 200 nm (LO = large oligomers) while later eluting fractions consist predominantly of short fibrils (MO = medium oligomers) of up to 100 nm and small, circular aggregates (SO = small oligomers) that can be smaller than 10 nm. Mature fibrils are generated from monomers by incubation at 37°C for 24 h and 300 rpm. We observed the same distribution of aggregates among the fractions with two different running buffers: 10 mM Tris–HCl, pH 7.4 or 100 mM Na-Borate, pH 8.6. These running buffers were chosen depending on the later purpose of the antigen. Aβ42 in 10 mM Tris buffer cannot be used for amine coupling of the antigen (e.g. in SPR experiments) while Aβ42 in 10 mM Tris–HCl, pH 7.4 is more suitable for immunization.

### Immunization and antibody phage display library construction

Late fractions of SEC purified Aβ42 oligomers (SO) were used for the immunization as well as for measuring the immune response by enzyme linked immunosorbent assay (ELISA). Ten days after the fifth boost, the antibody titer was determined to be 1 per 80,000. Nine weeks later a sixth boost was administered. PCR products of antibody genes were collected six and nine days after the last boost. The DNA fragments were pooled and subcloned into pGemT, resulting in a total of 2.7*10^6^ and 4.4*10^5^ individual clones for V_H_ and V_L_ respectively. pHAL35, a modified version of the pHAL14 phage display vector, was used for phage display library construction by two consecutive cloning steps. First, V_L_ gene fragments for the κ (kappa) and λ (lambda) were inserted using the restriction sites *Mlu*I and *Not*I followed by cloning of the V_H_ gene fragments via *Sfi*I and *Hind*III. The final libraries comprised a total of 2.9*10^7^ individual clones. The insert rates were determined by colony-PCR to be 60% for the kappa library and 80% for the lambda library. Both libraries were packaged using M13K07 as helperphage.

### Isolation of Amyloid-β specific scFvs

Multi-step pannings, with or without competition with unwanted forms of Aβ42 antigen (e.g. panning on immobilized fibrils with soluble monomers added for competition), were carried out to generate antibodies with diverse specificities against Amyloid-β. In addition to the two macaque IgG derived immune phage display libraries, two IgM derived naïve human phage display libraries HAL7/8 [44] were employed. From a total of 54 pannings, 6088 antibody clones were analyzed by ELISA and 612 hits were identified. Eight unique monoclonal antibodies with individual sequences, named PaD97-D6 from the naïve human libraries and PaD172-F8, PaD172-F12, PaD213-A5, PaD218-E6, PaD233-E5, PaD235-D2 and PaD236-H2 from the immune libraries were selected (Table 1) based on their specificity, their above average absorption or because of their high signal to noise ratio in the screening ELISAs. All eight antibodies were produced as scFvs and scFv-Fc fusions (Yumabs) [45] in mammalian cell culture. PaD172-F8, PaD218-E6 and PaD235-D2 could not be produced properly and disregarded for the following experiments.

**Table 1:** **Antibody pannings**

	**Libraries**		
	**HAL7/8**	**Aβ libraries (λ + K)**	**Total**
Number of pannings	28	26	54
Clones investigated	3236	2852	6088
Hits	2	610	612
Unique binders	1	7	8

Yumabs consist of a human IgG1 Fc part that is linked with two scFvs instead of Fab-fragments.

**Figure 2:**
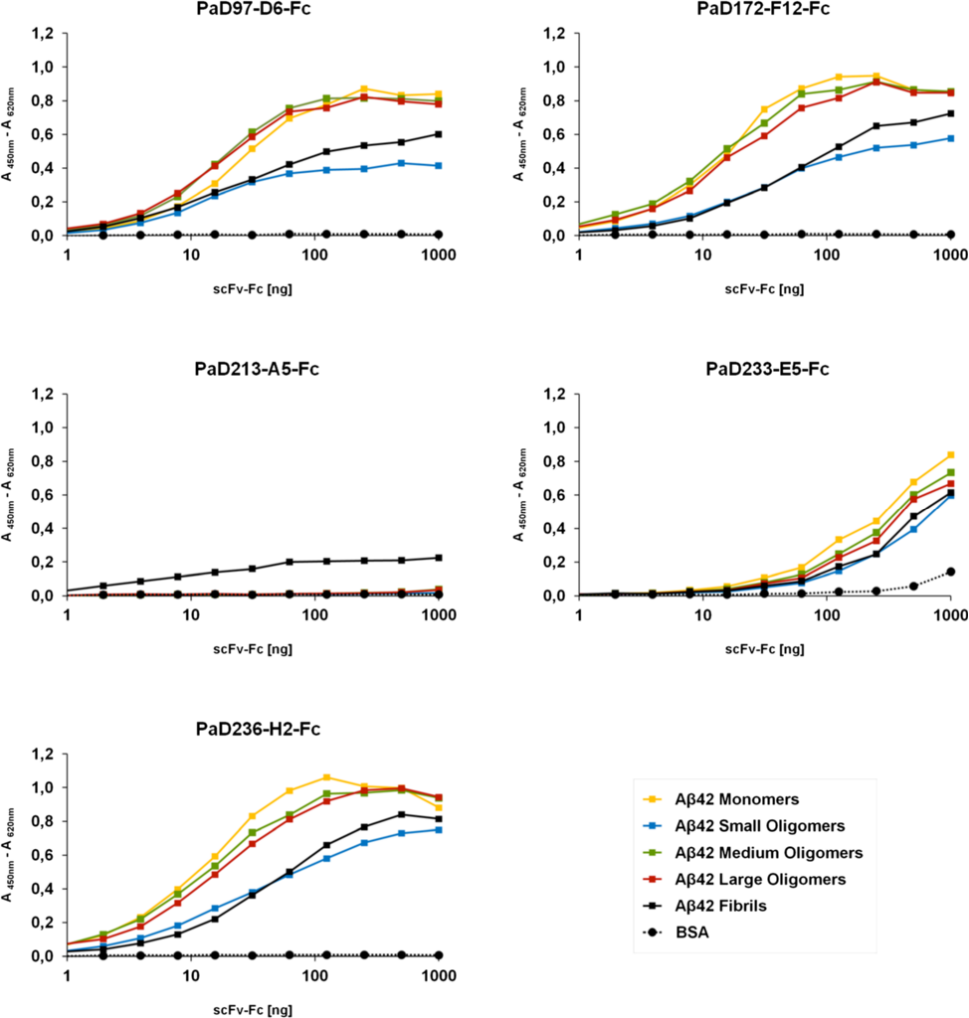
Specificity determination of scFv-Fc antibodies on 100 ng of Aβ42 monomers (yellow), small oligomers (blue), medium oligomers (green), large oligomers (red), fibrils (black) or BSA (dotted black). Dilutions of scFv-Fc antibodies were applied and detected using a peroxidase-labeled goat anti-human antibody recognizing the Fc fragment (1:35,000).

The specificity of PaD97-D6, PaD172-F12, PaD213-A5, PaD233-E5 and PaD236-H2 was initially verified on different forms of Aβ42, i.e. monomers, small, medium and large oligomers and mature fibrils by ELISA (Figure 2). Here, all antibodies except PaD213-A5 showed no predominant binding to any distinct form. Only PaD213-A5 exhibited specificity towards Aβ42 fibrils. Additionally, binding to fibrils of other amyloidogenic peptides was evaluated in the same manner. These peptides included mature fibrils of Aβ40, α-synuclein, Huntingtin (Htt (aa105-138)) and fibrils of Tau (isoform F), the K18 domain and the PHF6 domain of Tau. PaD97-D6 exhibited some cross reactivity with Tau fibrils of the isoform F (data not shown).

### PaD213-A5 differentiates between various Aβ42 fibrils

**Figure 3:**
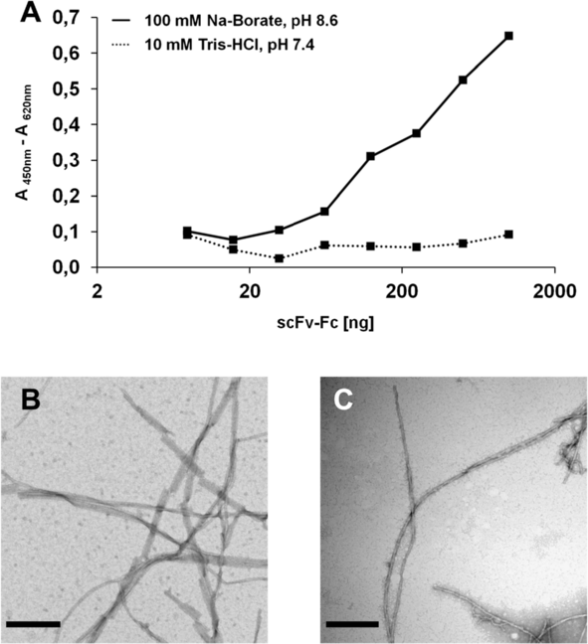
PaD213-A5 discriminates between different Aβ42 fibrils. **A**, titration ELISA of PaD213-A5 on different kinds of Aβ42 fibrils. Plates were coated with 100 ng of Aβ42 fibrils from 100 mM Na-Borate, pH 8.6 (solid line) or 10 mM Tris–HCl, pH 7.4 (dotted line). Bound ScFv-Fc antibodies were detected using a peroxidase-labeled goat anti-human antibody recognizing the Fc fragment (1:35,000). Right: representative TEM images of Aβ42 fibrils obtained from monomers purified in **(B)** 100 mM Na-Borate, pH 8.6 or **(C)** 10 mM Tris–HCl, pH 7.4, the scale bar corresponds to 200 nm.

Aβ42 peptide was purified in two different running buffers, 10 mM Tris–HCl, pH 7.4 or 100 mM Na-Borate, pH 8.6, depending on its later application. Repeated immunological assays elucidated the selectivity of PaD213-A5 towards a distinct form of Aβ42 fibrils. This antibody exhibited no affinity to mature fibrils produced in Tris–HCl buffer while on the other hand binding to fibrils generated in Na-Borate buffer (Figure 3A). TEM investigation revealed major differences in the composition of the fibrils. Na-Borate derived fibrils exhibited a compact bundle of 4–8 individual fibrils twisted helically every 130–150 nm (Figure 3B) while Tris derived fibrils consisted of one discrete fibril with a helical twist around its axis about every 50 nm (Figure 3C).

### All antibodies detect different epitopes

**Figure 4:**
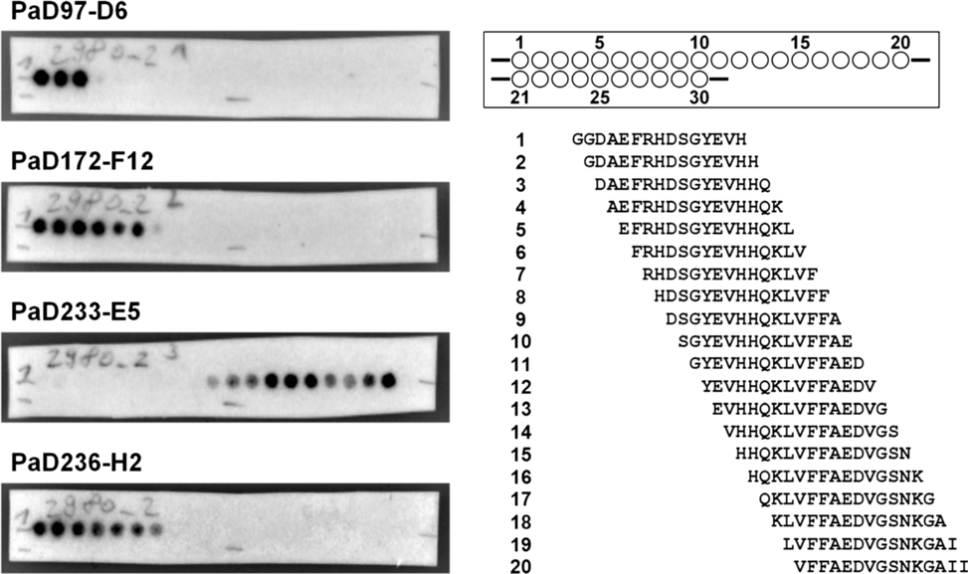
Epitope mapping analysis. Left: Membranes covering the complete sequence of Aβ42 with peptides of 15 amino acids in length and an offset of 1 amino acid. 10 μg/mL of scFv-Fc antibodies were used for staining, antibodies were detected using a peroxidase-labeled goat anti-human antibody recognizing the Fc fragment (1:70,000) PaD213-A5 not shown (no signal seen anywhere). Right: peptide spot map.

**Figure 5:**
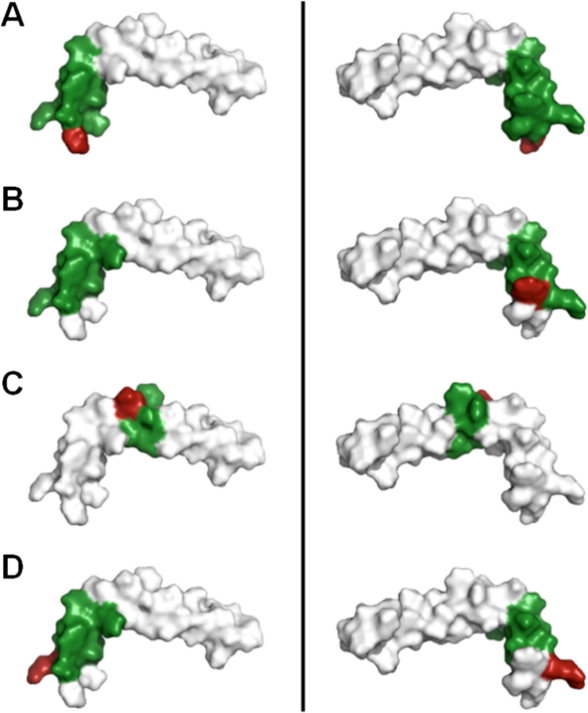
Visualization of the epitopes on Aβ42 on PDB structure 1z0q (Aβ42 monomer in aqueous solution, [78]). Left, right: same molecule rotated by 180° horizontally. Areas of the potential epitopes for **(A)** PaD97-D6, **(B)** PaD172-F12, **(C)** PaD233-E5 and **(D)** PaD236-H2 are colored green, the first amino acid of each epitope is colored red to facilitate recognition.

The determination of the epitope of the Amyloid-β specific antibodies was performed using a peptide spot membrane (Figure 4). Each spot on the membrane consisted of 15 AA of the Aβ42 peptide with an offset of 1 AA. Epitope mapping was performed with all antibodies to verify binding to linear epitopes. No binding was detected with PaD213-A5 since it is fibril specific, i.e. detecting a conformational epitope. PaD97-D6, PaD172-F12 and PaD236-H2 bound to the N-terminus of Aβ42 albeit differing in the exact epitope with position 1 to 13 for PaD97-D6 (“DAEFRHDSGYEVH”), position 4 to 13 for PaD172-F12 (“FRHDSGYEVH”) and position 5 to 13 for PaD236-H2 (“RHDSGYEVH”). A more precise determination of the epitopes for these three antibodies was impeded by the spot sizes of 15 AA in length. PaD233-E5 bound to the central region of Aβ42. Here, the exact epitope was more narrowly determined by amino acids 17 to 22 (“LVFFAE”) (Figure 5).

### Affinity determination of the scFvs by surface plasmon resonance (SPR)

Affinity determination was carried out on various Amyloid-β monomers, protofibrils and fibrils, via BIAcore™ with different antibody concentrations and resulted in K_D_ values in the micro- to nanomolar range (Table 2). The antibodies targeting the aminoterminal end of Aβ42 and each antibody, PaD97-D6, PaD172-F12 and PaD236-H2, exhibited similar affinities towards all three forms of antigen. In contrast, PaD233-E5 which binds to the core region of Aβ42 shows an 100-fold elevated affinity to Aβ42 monomers, with a K_D_ of 10 nM, when compared to protofibrils and fibrils. PaD213-A5 bound solely to Aβ42 fibrils with a K_D_ of 36 μM.

**Table 2:** **Affinity determination by SPR**

	**K**_**D**_ **[M] on Aβ42 antigen**		
**Antibody**	**Monomers (** ***X*** ^**2**^ **)**	**Protofibrils (** ***X*** ^**2**^ **)**	**Fibrils (** ***X*** ^**2**^ **)**
PaD97-D6	2.3 × 10^−6^ (4.9%)	4.2 × 10^−6^ (2.4%)	9.1 × 10^−7 ^(2.3%)
PaD172-F12	9.4 × 10^−7^ (2.4%)	1.5 × 10^−6^ (1.7%)	9.2 × 10^−7^ (2.4%)
PaD213-A5	No binding	No binding	3.7 × 10^−6^ (1.1%)
PaD233-E5	1.0 × 10^−8^ (6.6%)	3.6 × 10^−6^ (1.4%)	1.2 × 10^−6^ (1.4%)
PaD236-H2	3.5 × 10^−7^ (2.5%)	5.8 × 10^−7^ (1.9%)	6.2 × 10^−7^ (5.7%)

Chi^2^ (*Χ* ^**2**^) values are indicated in brackets.

### Yumabs inhibit Aβ42 fibrillogenesis in a concentration dependent manner

**Figure 6:**
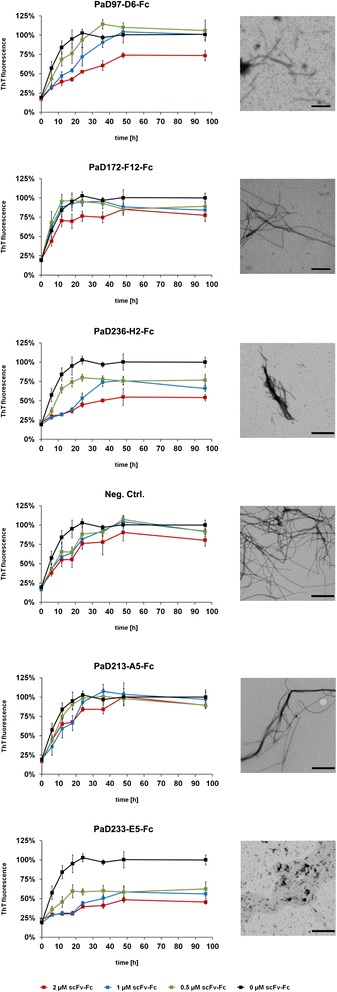
Influence of scFv-Fc antibodies (Yumabs) on Aβ42 fibrillogenesis. Left: 5 μM Aβ42 monomers were incubated with 2 μM (red), 1 μM (blue), 0.5 μM (green) or 0 μM (black) of scFv-Fc antibodies at 37°C under constant agitation of 300 rpm, ThT fluorescence was monitored over a time course of 96 h. All measurements were carried out in triplicates, the error bars represent the respective standard deviation. Right: representative TEM images of the fibrils formed from of 5 μM Aβ42 monomer after 96 h incubation in the presence of 2 μM scFv-Fc antibody, scale bar corresponds to 400 nm.

When binding to Aβ42 monomers, an inhibitory effect of the antibodies on fibril formation could be possible. We tested the effect of all antibodies on the formation of mature Aβ42 fibrils from pure monomers by visualizing potential fibrils using transmission electron microscopy (TEM) and measuring Thioflavin T (ThT) fluorescence. ThT is a dye that, upon binding to amyloid fibrils, exhibits fluorescence. Thus it allows for the assessment of fibril formation, which was investigated in this study by combining part of the sample with ThT stock solution every six hours during the first 24 h, every 12 h during the next 24 h and with a final checkpoint after 96 h (Figure 6).

Bivalent scFv-Fc antibodies (Yumabs) were able to interfere with fibril formation at a substoichiometric level for PaD97-D6, PaD233-E5 and PaD236-H2 (Figure 6). The influence is most notable for PaD233-E5, the antibody targeting the central α-helical region of Aβ42. Addition of 4 μM scFv-Fc antibody to 5 μM Aβ42 monomers resulted in a reduction in ThT fluorescence of about 25% for PaD97-D6, nearly 50% for PaD236-H2 and even more elevated forPaD233-E5 after 96 h of incubation (Figure 6). Comparison with PaD213-A5 or the negative control scFv-Fc antibody indicates that this effect is not contributed to antibody concentration or design. Interestingly, PaD172-F12, also directed against the N-terminal end of Aβ42 like PaD97-D6 and PaD236-H2, did not show an inhibitory effect.

The reverse mechanism, a disintegration of preformed fibrils by antibody addition, was evaluated by ThT reading and TEM analysis as well. No antibody mediated disintegration of mature fibrils (data not shown).

## Discussion

Aβ42 oligomers were chosen for the immunization of the NHP due to their reported elevated toxicity, making them a potential target for immunotherapy. Using the immune libraries and two previously established human naïve libraries [32] in a multistep panning, we created numerous antibody fragments specific for β-amyloid with an interesting spectrum of different binding properties.

The initial validation utilizing titration ELISAs demonstrated antibody specificity towards either form of Aβ42 aggregates but no predominant preference for PaD97-D6, PaD172-F12, PaD233-E5 or PaD236-H2. Epitope mapping further revealed that three of these four antibodies detect the N-terminal part of Aβ42 whereas PaD233-E5 binds to the central region. This is consistent with previous findings that the amino-terminal region of Aβ42 is immunodominant in human [46], NHP [47] as well as in dog [48], mouse [49] and rabbit [50] explaining the quantity of antibodies and antibody fragments directed against this part of the peptide in this work and previous studies [49,51-55], with Bapineuzumab being the most prominent one. Solely PaD213-A5 demonstrated a high selectivity towards Aβ42 fibrils and did not bind to any other form of Aβ42. Remarkably, PaD213-A5 was able to even discriminate between two different Aβ42 fibril preparations, depending whether the amyloid-β peptide was purified in 10 mM Tris–HCl/pH 7.4 or in 100 mM Na-Borate/pH 8.6.

Meinhardt et al. [56] already described other preparation dependent polymorphisms in Aβ40 fibrils. Based on their findings, it seems likely that the difference in the acidity of the buffers contributes to a morphological change in the fibril structure, a hypothesis that is supported by our TEM analysis. It can be hypothesized that PaD213-A5 distinguishes between both types of fibrils through the detection of a conformational epitope which may well be dependent on the helical twist angle or the interspace distances between two single strands that make up the mature β-amyloid fibril. While there are antibodies and polyclonal sera that are fibril specific [57-59] the specificity observed here was not reported for any other known antibody. It remains to evaluated whether these structural differences have any significance *in vivo*.

To investigate the antibodies for a potential application as disease modulators, we assessed their impact on the fibrillization of Aβ42 monomers *in vitro*. The fibrillogenesis of Aβ42 is a nucleation-dependent polymerization process [8]. When a certain concentration threshold of monomers is surpassed small aggregates termed “nuclei” accrue and polymerization starts. These nuclei are elongated by addition of monomers forming larger aggregates and ultimately fibrils. It has been previously shown that antibodies targeting the N-terminal end of amyloid-β exhibit an inhibitory effect on the fibrillogenesis [60-63]. With the majority of our antibodies recognizing Aβ42 monomers this gives rise to the idea that they can intervene in the initial aggregation by preventing interactions of β-amyloid peptides thus retarding or even inhibiting fibril formation [64].

PaD97-D6 and PaD236-H2 demonstrate a concentration dependent retardation of fibril formation resulting in shorter fibrils and an overall stronger appearance of unstructured aggregates. They do not prevent fibrillization entirely, which suggests a steric hindrance during monomer-monomer attachment [61]. Albeit also binding to the amino-terminal region of Aβ42, PaD172-F12 exhibited no substantial effect on fibril formation. With no major discrepancies in the affinity compared to PaD97-D6 or PaD236-H2, this result is likely to be accounted to the minute differences in epitopes. It is plausible that PaD172-F12 attaches to monomers in such way that no steric hindrance is administered towards the core region of Aβ42. A partial masking of that area by an antibody would minimize monomer-monomer interaction and impede fibril formation. Epitope mapping demonstrates that PaD97-D6 binds Aβ_1–13_ while PaD172-F12 and PaD236-H2 bound further downstream (Aβ_4–13_ for PaD172-F12 and Aβ_5–13_ for PaD236-H2). Obviously, PaD97-D6, PaD172-F12 and PaD236-H2 attach to the monomer with different spatial arrangements. Further, the region of the epitope on the Aβ42 peptide may contribute to the similar K_D_ values to different aggregates measured for these antibodies. PaD97-D6, PaD172-F12 and PaD236-H2 bind to the amino-terminal end of the β-amyloid peptide, an epitope that is exposed in monomers and aggregates during fibrillogenesis [54]. This may allow nearly equal affinities of the before mentioned antibodies to all three forms.

PaD233-E5 impacts fibril formation, which is not surprising as it targets the central region of Aβ42 with Aβ_17–22_ (LVFFAE), a part of the hydrophobic core element (LVFF) that is essential for β-sheet formation during fibrillization [65]. Together with the elevated affinity towards Aβ42 monomers, this effect can be accounted to two probable modes of action or a mixture of both. PaD233-E5 either masks the LVFF-motif thus directly preventing monomer-monomer interaction. This effect was postulated by Legleiter et al. for the antibody m266, the murine progenitor of Solanezumab [61]. m266 targets the same epitope as PaD233-E5, binding to Aβ_16–24_ (KLFFAEDV) [66] and prevents the formation of fibrils and even protofibrils. The other possible explanation is the attachment of PaD233-E5 to Aβ42 monomers thus shifting the concentration threshold of soluble β-amyloid beneath the critical limit necessary for the polymerization process. Interestingly, PaD233-E5 has a much more pronounced influence on amyloid-β fibrillogenesis than any other antibody as visualized by TEM. Yet the ThT absorbance after 96 h is similar to that of PaD236-H2 which might be an indication for the formation of smaller aggregates with a β-sheet rich content. This would suggest the latter mode of action described for PaD233-E5 to be more dominant in the inhibition process.

The impact on AD immunotherapy of the antibodies presented in this work has to be further validated. Recently, Bapineuzumab (directed against the N-terminus of Aβ) and Solanezumab (directed against the central region of Aβ), both not conformation specific antibodies, failed to meet the expected endpoints in clinical phase 3 studies albeit having shown positive results in preceding studies (reviewed in [67]). The results of the initial characterization for the Yumabs in this work are promising. Especially PaD213-A5 exhibits a highly interesting property of differentiating between Aβ42 fibrils based on their conformation that is not yet described in literature and its implication on AD diagnosis and therapy has to be further validated with *in vivo* data.

## Conclusion

Among the investigated antibody fragments we found three scFvs exhibiting a general specificity towards β-amyloid while two scFvs, PaD213-A5 and PaD233-E5, presented a tendency to better bind to certain forms of Aβ42. PaD213-A5 is highly specific for mature Aβ42 fibrils and identified a novel structural variation in fibrillar structures. PaD233-E5, albeit binding also oligomers and fibrils, showed a 100fold increased affinity towards monomers. It is also one of the three antibodies exhibiting an inhibitory effect on the fibrillization of Aβ42 monomers.

While the *in vivo* relevance of these differences is still to be established, the study confirms that the approach of animal immunization and subsequent phage display based antibody selection is applicable to generate highly specific anti β-amyloid scFvs that are capable of accurately discriminating between minute conformational differences.

## Methods

### Antigen preparation

Aβ42 peptides were synthesized by Dr. James I. Elliott at Yale University (New Haven) [68]. All Aβ42 antigens, including monomers, protofibrils and different size oligomers derived thereof by further fractionation as well as Fibrils were prepared according to [18,69].

### TEM sample grid preparation and image acquisition

5–10 μL of sample was deposited on a formvar coated 200 mesh TEM grid (EM Science, Hatfield) and incubated for 1 min. Excess fluids were wicked away with a piece of filter paper. The grid was washed twice by applying 10 μL of dH_2_O before incubating the sample twice with 10 μL of 2% (w/v) uranyl acetate for 1 minute each. The grid was dried with a vacuum pump, incubated for 5 min at room temperature to completely dry off and stored in the designed container. Imaging was carried out on a Tecnai G2 Spirit microscope at an acceleration voltage of 80 kV.

### Ethics statement and animal care

All animal studies presented were given specific approval from the *Institut de Recherche Biomédicale des Armées* ethics committee (*Comité d'éthique de l'Institut de Recherche Biomédicale du Service de Santé des Armées*) under authorization no. 2008/03.0 and were performed in accordance with all relevant French laws and ethical guidelines, including, in particular (i) “partie règlementaire du livre II du code rural (Titre I, chapitre IV, section 5, sous-section 3: expérimentation sur l’animal)”, (ii) “décret 87–848 du 19-10/1987 relatif aux expériences pratiquées sur les animaux vertébrés modifié par le décret 2001/464 du 29/05/2001”, (iii) “arrêté du 29 octobre 1990 relatif aux conditions de l’expérimentation animale pour le Ministère de la Défense” and (iv) “instruction 844/DEF/DCSSA/AST/VET du 9 avril 1991 relative aux conditions de réalisation de l’expérimentation animale”.

Animal care procedures complied with the regulations detailed under the Animal Welfare Act [70] and in the Guide for the Care and Use of Laboratory Animals [71]. Animals were kept at a constant temperature (22°C+/−2°C) and relative humidity (50%), with 12 hours of artificial light per day. They were housed in individual cages (6 per room), each of which contained a perch. Animals were fed twice daily, once with dried food and once with fresh fruits and vegetables, and water was provided at the same time. Food intake and general behavior were observed by animal technicians during feeding times, and veterinary surgeons were available for consultation if necessary. Veterinary surgeons also carried out systematic visits to each NHP-room twice weekly. The environmental enrichment program for the nonhuman primates was limited to games with animal care staff and access to approved toys. The well-being of the animals was monitored by the attending veterinary surgeon. Animals were anesthetized before the collection of blood or bone marrow by an intramuscular injection of 10 mg/kg ketamine (Imalgene®, Merial, Lyon, France). Analgesics were subsequently administered, through a single intramuscular injection of 5 mg/kg flunixine (Finadyne®, Schering Plough, Courbevoie, France) in the days after interventions if the animal technicians suspected that the animal was in pain, on the basis of their observations of animal behavior. None of the nonhuman primates were killed during this study.

### Animal immunization

A male macaque (*Macaca fascicularis*) was immunized with a total of 6 subcutaneous injections of purified and sterile filtered small oligomers of Aβ42. Injections were carried out with 50 μg antigen (inj. 1–3) and 50 μg antigen (inj. 4–6) at a one month interval, except for the sixth injection which was given 2 months after the fifth.

### Construction of the anti Aβ42 scFv phage display library

Six and nine days respectively after the last boost, RNA was isolated using Tri Reagent (Molecular Research Center Inc, Cincinnati, USA) from the bone marrow of the immunized macaque and transferred into cDNA by reverse transcription. DNA was amplified by PCR using seven different oligonucleotide primers for the coding regions of the light chain and nine different primers for the heavy chain [72]. After amplification, PCR products were pooled and subcloned into pGemT (Promega, Madison, Wisconsin). Antibody inserts in pGemT were re-amplified with individual primer sets for the kappa (κ) and lambda (λ) sublibraries introducing specific restriction sites for the cloning of the final library as described [44]. Library packaging was carried out using M13K07 as helperphage.

### Selection of recombinant antibodies against Aβ42

ScFvs were isolated *in vitro* by panning the macaque derived immune libraries as well as the human naïve libraries HAL7/8 [44] as described previously [73]. Antigen coating was carried out at 4°C overnight in 100 mM Na-Borate buffer and constant amounts (1 μg) of antigen were used as bait during the three panning rounds. To increase the possibility of obtaining antibodies specific for one Aβ42 conformation, competition with unwanted conformations of Aβ42 was done using 3 μg of antigen or 5 μg for Aβ42 fibrils respectively. Individual colonies of bacteria infected with eluted antibody phage were isolated and inoculated in MTP (microtiter plate) wells to produce soluble antibody fragments as described previously [74]. The produced scFvs were analyzed for specific binding by ELISA on diverse aggregates of Aβ42, corresponding to the panning.

### Enzyme linked immunosorbent assay (ELISA)

Two kinds of ELISA (screening ELISA, antigen titration ELISA) were performed as described before [74]. In both cases a total of 100 ng of antigen per cavity was coated in 96well MTPs (High Binding, Costar) at 4°C overnight. All following steps were carried out at room temperature on a rocker. For screening, scFvs were detected by mAb 9E10, recognizing the c-myc tag and a goat anti-mouse antibody conjugated to horseradish peroxidase (Sigma A0168). For titration, scFvs were detected by a mouse anti penta-His (34660, Qiagen), recognizing the His tag and a goat anti-mouse antibody conjugated to horseradish peroxidase (A0168, Sigma Aldrich). Bound scFv-Fc antibodies were detected using a peroxidase-labeled goat anti-human antibody recognizing the Fc fragment (A0170, Sigma-Aldrich).

### ScFv and scFv-Fc antibody production and purification

The scFv inserts of positive clones were subcloned into the pCSE2.5-HIS-XP and the pCSE2.5-hIgG1-Fc-XP vector, and the resulting scFv or scFv-Fc antibodies (Yumabs) were transiently produced in HEK293-6E cells [75] as described previously [45]. The cultivation medium was chemically defined F17 medium (Invitrogen, Life Technologies) supplemented with 7.5 mM L-glutamine, 0.1% PF68 (Applichem) and 1% Penicillin/Streptomycine. Antibody fragments were purified using immobilized metal ion (Ni^2+^) or protein A affinity chromatography on the Profinia™ Affinity Chromatography Protein Purification System (BioRad), according to the manufacturer’s protocol.

### Thioflavin T (ThT) measurements

To assess the state of fibrillogenesis by Thioflavin T (ThT) measurement, 20 μL of sample was mixed with 10 μL of ThT (100 μM) and 70 μL of glycine NaOH, pH 8.5 (500 mM) in a well of a black 384-well Nunc plate (Sigma-Aldrich). Fluorescence was measured in triplicates on an Analyst™ AD fluorometer (Molecular Devices Cooperation) at an excitation wavelength of λ = 450 nm and emission wavelength of λ = 485 nm.

### Epitope mapping

The peptide sequence of Aβ42 was divided into overlapping peptide fragments of 15 aa length with an offset of 1 aa. The N-terminus was acetylated and two additional glycines were added to the sequence to allow for proper binding of the antibodies to the aspartic acid, the first aa of Aβ42. The peptides were synthesized by the SPOT technique [76,77] and covalently bound to a continuous cellulose membrane via their carboxy-terminus (JPT Peptide Technologies GmbH). After initial incubation for 5 min in methanol to prevent the precipitation of hydrophobic peptides the membrane was rinsed with 1xTBS (50 mM TRIS, 137 mM NaCl, 2.7 mM KCl, pH adjusted to 8.0 with HCl) and blocked in 2% (w/v) skim milk powder in 1xTBS (2% M-TBS) for 1 h at room temperature on a rocker. ScFv-Fc antibodies (10 μg/mL in 2% M-TBS) were incubated on the membranes for 1.5 h at room temperature. Bound antibodies were detected by using a peroxidase-labeled goat anti-human antibody recognizing the Fc fragment (A0170, Sigma-Aldrich). Development with SuperSignal West Pico Chemiluminescent Substrate (Thermo Scientific) according to manufacturer’s protocol on a ChemiDoc™ MP system (BioRad).

### Affinity measurement

Antibody affinities were analyzed by surface plasmon resonance (SPR) using a BIAcore2000™. Aβ42 monomers and protofibrils were immobilized on separate CM5 chips (General Electric-Biacore), fibrils were immobilized on a CMD50m chip (Xantec) via amine coupling according to the manufacturers protocols. ScFvs were diluted to 100 nM - 10,000 nM (additionally to 15,000 nM for PaD97-D6 and 15,000 nM + 20,000 nM for PaD213-A5) and added to the chips in HBS-EP buffer according to the manufacturer’s protocol at a flow rate of 25 μL/min. Timeframes were 200 s for association and 600 s for dissociation. After each dilution, the chip was regenerated with NaOH according to the manufacturer’s protocol. Data fitting was performed using the “1:1 binding with drifting baseline” algorithm of the BIAevaluation™ software.

## Abbreviations

aa: Amino acid
Aβ: Amyloid-β/β-amyloid
AD: Alzheimer’s disease
NHP: Non human primate
scFv: Single chain fragment variable
Fc: Fragment crystallizable
TEM: Transmission electron microscopy
ThT: Thioflavin T
